# Circulating Tumor DNA in Merkel Cell Carcinoma: A Precision Biomarker for Recurrence Detection and Therapeutic Guidance

**DOI:** 10.3390/jpm16060330

**Published:** 2026-06-20

**Authors:** Joshua E. Chan, Lisa C. Zaba

**Affiliations:** Department of Dermatology, Stanford University School of Medicine, Stanford, CA 94305, USA; jechan@stanford.edu

**Keywords:** Merkel cell carcinoma, circulating tumor DNA, minimal residual disease, surveillance, precision oncology

## Abstract

**Background/Objectives:** Merkel cell carcinoma (MCC) is a rare but aggressive skin cancer with a 40% recurrence rate. However, reliable biomarkers for early recurrence detection or treatment guidance are lacking, especially for virus-negative tumors. Circulating tumor DNA (ctDNA), a fragment of tumor-derived cell-free DNA in blood, has emerged across multiple cancers as a minimally invasive precision biomarker to detect minimal residual disease (MRD); predict recurrence; and monitor treatment response. This review’s objective was to summarize recent advances in ctDNA as a tool for therapeutic decision-making in MCC, contextualized by findings in other malignancies. **Methods:** A comprehensive literature review was performed, focusing on studies published between 2016 and 2026 that evaluate ctDNA in MCC and other cancers. Key prospective trials, observational studies, and case reports were identified through PubMed and relevant conference proceedings. Data on ctDNA assay methods (tumor-informed vs. tumor-agnostic), clinical sensitivity, lead time for recurrence detection, and predictive value for therapy response were extracted and synthesized. **Results:** Across cancers such as colorectal, lung, and melanoma, ctDNA positivity after curative treatment predicts relapse months in advance of imaging and can guide adjuvant therapy decisions. In MCC, recent studies demonstrate that ctDNA levels correlate with MCC tumor burden and exhibit high sensitivity and specificity for clinically evident disease. Stage I-III MCC patients who were ctDNA-positive within four months of treatment had a 7.4-fold higher recurrence risk within the subsequent 12–18 months of follow-up. Serial ctDNA monitoring may enable earlier intervention in otherwise asymptomatic ctDNA-positive MCC cases, helping distinguish responders from non-responders. **Conclusions:** ctDNA is an emerging precision biomarker that offers significant prognostic and surveillance utility in MCC. It enables earlier detection of recurrence, potentially allowing treatment to begin before clinical disease manifests. It also helps stratify patients by risk and treatment response, informing personalized surveillance intensity and therapeutic choices. Integrating ctDNA monitoring into MCC management could improve outcomes by guiding timely interventions, although prospective trials are needed to confirm that ctDNA-guided decisions translate to improved patient survival. Formal cost-effectiveness analyses have not yet been conducted and represent an important area for future investigation.

## 1. Introduction

Merkel cell carcinoma (MCC) is an aggressive neuroendocrine skin cancer with a 40% recurrence rate [1,2]. Current follow-up relies mainly on physical exams and imaging, but these methods can only detect macroscopic disease and may miss early microscopic recurrence. Moreover, MCC lacks universally reliable blood biomarkers: in virus-positive cases (which comprise ~80% of MCC in the U.S.), patients can be monitored with Merkel polyomavirus oncoprotein antibody titers, but this approach is ineffective for the ~20% of virus-negative tumors and requires baseline titers [3]. Thus, there is a critical unmet need for a precision biomarker that can noninvasively detect residual or recurrent MCC across all patients and guide therapeutic decisions.

**Figure 1:**
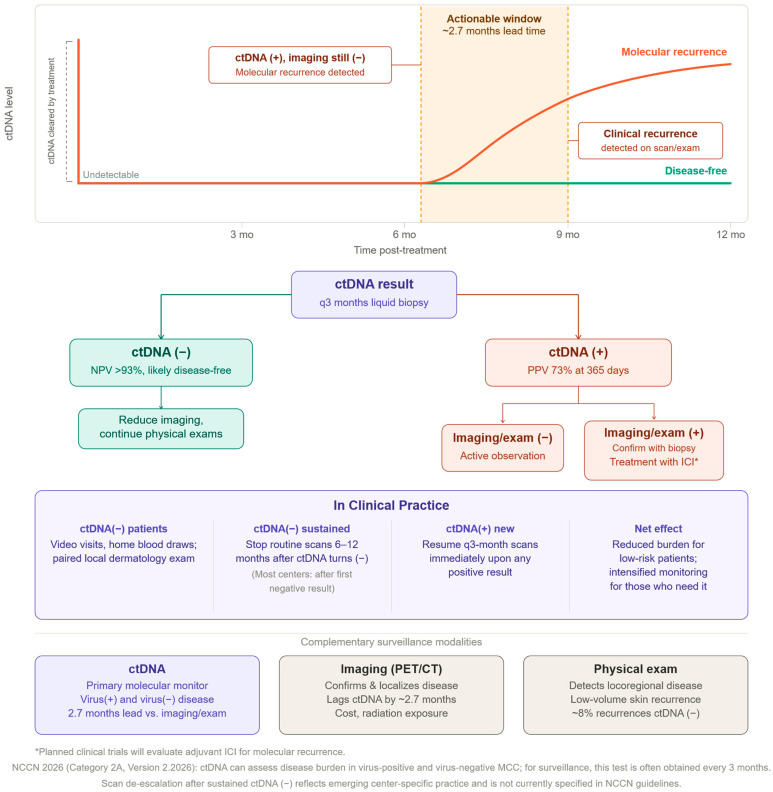
ctDNA-guided clinical management of Merkel cell carcinoma. (**Top**): Serial ctDNA levels following curative-intent treatment. (**Middle**): Decision algorithm based on ctDNA result. A negative result (NPV > 93%) supports imaging de-escalation and continued physical surveillance. A positive result prompts imaging/exam to distinguish active observation (imaging-negative) from biopsy-confirmed treatment (imaging-positive). (**Bottom**): Real-world practice implications and complementary roles of ctDNA, imaging, and physical examination. ctDNA: circulating tumor DNA; ICI: immune checkpoint inhibitor; NPV: negative predictive value; PPV: positive predictive value; MCC: Merkel cell carcinoma.

Circulating tumor DNA (ctDNA) has rapidly emerged as such a biomarker in oncology. ctDNA refers to fragments of double-stranded DNA shed from tumor cells into the bloodstream, typically shorter than non-tumor cell-free DNA and carrying tumor-specific genetic alterations. Its short half-life (approximately 16 min to 2.5 h) means ctDNA levels reflect real-time tumor burden [4]. Importantly, ctDNA can be analyzed via a simple blood draw (liquid biopsy), offering a minimally invasive alternative to repetitive biopsies or heavy reliance on imaging. Early studies showed strong concordance between mutations identified in tumor tissue and those detected in plasma ctDNA, validating that liquid biopsy captures the molecular profile of the cancer. Because tumors continuously release DNA, serial ctDNA measurements enable dynamic monitoring of disease status and treatment efficacy in ways that a snapshot tissue biopsy cannot. These features position ctDNA as a powerful tool in precision medicine to detect minimal residual disease, predict relapse, and tailor therapies based on molecular disease activity. The clinical integration of ctDNA into MCC surveillance and therapeutic decision-making is summarized in Figure 1.

Technological advances in sequencing have markedly improved ctDNA detection sensitivity in the last decade, making clinical applications feasible. Two broad strategies exist: tumor-agnostic assays and tumor-informed assays. Tumor-agnostic tests (e.g., Guardant360, FoundationOne Liquid) use predefined panels of commonly mutated genes and examine plasma directly for any mutations, without prior knowledge of a patient’s tumor genotype. While convenient, these panels may miss rare or patient-specific mutations and have limited sensitivity when tumor DNA fractions are very low. In contrast, tumor-informed approaches first sequence each patient’s tumor (and often normal DNA) to identify a set of unique somatic mutations, then design a personalized assay (e.g., Natera’s Signatera) to track those mutations in the patient’s plasma with ultra-deep sequencing. Overall, these technical innovations have paved the way for ctDNA to serve as a more sensitive and personalized biomarker in solid tumors.

This review focuses on the clinical utility of ctDNA in MCC, with context drawn from other malignancies where ctDNA has been evaluated as a precision biomarker. We first overview how ctDNA is being used across cancers for MRD detection and treatment guidance. We then highlight key findings from recent MCC studies that demonstrate the high prognostic value of tumor-informed ctDNA assays for detecting recurrence and guiding management. Finally, we discuss the implications for clinical practice, current limitations, and future directions needed to fully integrate ctDNA-guided care in MCC.

## 2. Methods

A comprehensive literature review was performed to identify studies evaluating ctDNA in MCC and other solid tumors. PubMed and relevant oncology conference proceedings (ASCO, AACR, AAD, SID) were searched for articles published between January 2016 and January 2026 using the following keywords: “circulating tumor DNA,” “ctDNA,” “liquid biopsy,” “Merkel cell carcinoma,” “minimal residual disease,” “tumor-informed assay,” and “tumor-agnostic assay.” Studies were included if they reported on ctDNA detection methods, clinical sensitivity or specificity, prognostic outcomes, or treatment guidance in MCC or in analogous solid tumors (colorectal cancer, melanoma, non-small cell lung cancer, breast cancer). Case reports were included when they provided unique mechanistic or clinical insights. Non-English language studies and conference abstracts without accompanying peer-reviewed data were excluded.

## 3. ctDNA as a Precision Biomarker Across Cancers

Over the past decade, ctDNA has been investigated in numerous cancer types as a tool for risk stratification and therapy guidance. Post-operative MRD detection is one major application: in several solid tumors, the presence of ctDNA in plasma after curative-intent surgery or treatment is a strong predictor of eventual relapse. For example, in colorectal cancer, ctDNA-guided adjuvant therapy is an area of active research. Henriksen et al. monitored serial ctDNA in 168 stage III colorectal cancer patients and found that patients who cleared ctDNA after surgery and adjuvant chemotherapy had no recurrences, whereas 80% patients with persistent ctDNA relapsed [5]. Similarly, the GALAXY study (part of the CIRCULATE-Japan trial) reported that, among ctDNA-positive patients with resectable colorectal cancer who did receive adjuvant chemotherapy, the recurrence rate was ~61% [6]. Meanwhile, those who were ctDNA-positive but did not receive adjuvant chemotherapy had a nearly universal recurrence rate at 95.74%. The “spontaneous” clearance rate (clearing ctDNA without any chemotherapy) was extremely rare, occurring in only ~2% of cases. These data illustrate how ctDNA can identify patients who harbor residual disease and are likely to benefit from additional therapy, while ctDNA-negative patients might safely avoid unnecessary treatment. In early-stage non-small cell lung cancer, ctDNA has similarly shown prognostic value: detection of ctDNA after surgery or definitive therapy strongly correlates with recurrence risk. For instance, one study found ctDNA was detectable in 93% of relapsed NSCLC cases and signaled relapse with a median lead time of ~70 days (over two months) before radiographic detection [7]. This lead time offers a critical window during which early intervention might be initiated. Breast cancer studies likewise indicate that ctDNA-based MRD detection can foretell metastasis months before clinical relapse, allowing for consideration of pre-emptive systemic therapy [8].

Beyond predicting “if” and “when” a recurrence will occur, ctDNA is proving useful to monitor treatment response and resistance in real time. In metastatic melanoma and other immunogenic tumors, changes in ctDNA levels correlate with response to immune checkpoint inhibitors (ICIs). A recent meta-analysis of over 1000 melanoma patients on ICIs showed that patients with detectable ctDNA during treatment had a significantly higher risk of poor outcomes (OS HR = 4.57; PFS HR = 3.79) compared to pre-treatment levels, showing that the persistence of ctDNA is a real-time signal that the therapy is not clearing the malignancy [9]. Persistent or rising ctDNA often portends primary resistance to immunotherapy, whereas clearance or large decreases in ctDNA after a few weeks of therapy are associated with favorable responses. These findings suggest ctDNA can serve as an early indicator of whether a treatment is effective, well before traditional imaging might show tumor shrinkage or growth. In diseases like advanced lung cancer, serial ctDNA measurements have revealed the emergence of resistance mutations (e.g., EGFR T790M) months prior to radiographic progression, enabling timely switch to appropriate targeted therapies [10]. In summary, across multiple malignancies, ctDNA has demonstrated value for real-time surveillance: detecting microscopic disease that remains after treatment, forecasting relapse earlier than standard methods, and informing adjustments to therapy (escalation, switching, or de-escalation) based on molecular response.

These advances across cancers establish the paradigm that liquid biopsies can personalize therapy timing and selection. MCC shares challenges common to aggressive solid tumors: a high relapse rate after initial treatment and variable responses to systemic therapy (notably immunotherapy). The next sections focus on how ctDNA is being applied in MCC to meet these challenges, building on the principles proven in other cancers.

## 4. Merkel Cell Carcinoma: Disease Characteristics and Biomarker Needs

MCC is characterized by an aggressive clinical course and an immunogenic biology. Around 80% of cases are driven by the Merkel cell polyomavirus (MCPyV), while the remainder are UV-induced, virus-negative tumors with a high mutation burden. Despite different etiologies, all MCC patients require close surveillance due to the cancer’s tendency to recur locally, regionally, or distantly even after initial treatment. Standard of care involves frequent imaging (e.g., CT or PET scans) and physical exams, especially in the first 2–3 years post-treatment when most recurrences occur. However, this routine is one-size-fits-all, and imaging every patient on a fixed schedule leads to many costly scans, anxiety, and radiation exposure, often without yielding actionable findings. A more individualized approach to surveillance intensity—guided by a biomarker indicating which patients are likely or unlikely to relapse—could greatly enhance care.

Previously, the only circulating biomarker utilized in MCC management was the anti-MCPyV oncoprotein antibody titer (AMERK), and only for the subset of patients with virus-positive tumors. Patients whose tumors express MCPyV T antigens often mount an immune response, producing circulating antibodies. These antibody levels tend to fall after complete tumor resection and rise again if tumor burden returns, making them a useful proxy for disease status. Indeed, rising oncoprotein titers have been associated with impending recurrence in virus-positive MCC, and checking these titers periodically can help signal relapse or monitor response to therapy. However, there are critical caveats: titers must be interpreted relative to an individualized baseline soon after initial diagnosis (since patients are seropositive or seronegative with varying baseline levels), and about 20% of MCC patients are seronegative even if their tumor is virus-driven. Moreover, MCPyV-negative MCC cannot be monitored this way at all, and these UV-associated tumors lack any equivalent blood marker. Even among virus-positive cases, antibody tests are imperfect, as some MCC patients never develop a measurable titer. Therefore, while viral serology is a valuable tool, it is not a comprehensive solution for MCC surveillance or treatment guidance.

In this context, ctDNA has offered a promising universal biomarker for MCC, applicable to both MCPyV-positive and -negative cases. Because ctDNA directly reflects tumor-derived genetic material, it is not dependent on viral status. Furthermore, MCC tumors often harbor recurrent mutations in genes like TP53, RB1, and others, providing molecular targets that a ctDNA assay can track. Traditional tissue biomarkers such as PD-L1 expression or total tumor mutational burden (TMB) have not reliably predicted outcomes in MCC in prospective studies, limiting their clinical utility for treatment selection, and other investigated serum markers (like neuron-specific enolase) have shown inconsistent results. Thus, a ctDNA-based approach could fulfill a major unmet need: a sensitive, tumor-specific indicator of disease burden that could change how clinicians monitor MCC patients and make therapeutic decisions, such as starting adjuvant therapy and intensifying or sparing interventions.

## 5. Clinical Adoption of ctDNA for MCC Surveillance

When considering whether to adopt ctDNA clinically, it is important to evaluate evidence that it truly performs well in MCC. Key questions include: Can ctDNA detect residual or recurrent MCC earlier than current methods? Does ctDNA positivity correlate with higher recurrence risk, and conversely, does negativity signal a low-risk state? Can ctDNA measurements inform or predict response to therapies like immunotherapy? Below, we review the growing body of literature that addresses these questions in MCC.

### 5.1. NCCN Guideline Integration

The clinical landscape for MCC shifted significantly with the release of the NCCN Guidelines Version 1.2026, which officially integrated ctDNA into the management algorithm for the first time [11]. Recognizing the limitations of viral serology, the NCCN now recommends ctDNA as a formal tool for surveillance and workup (Category 2A). For patients in the surveillance phase, the guidelines suggest obtaining ctDNA testing. While a specific surveillance interval is not mandated, emerging academic center practice commonly employs ctDNA testing every 3 months during the first 2–5 years post-treatment, aligning with the window of highest recurrence risk. The NCCN acknowledges that ctDNA can assess disease burden in both virus-positive and virus-negative MCC, providing the first standardized blood-based biomarker for the approximately 20% of patients with UV-driven tumors who were previously “invisible” to circulating molecular monitoring.

### 5.2. ctDNA Versus MCPyV Antibody Titers

Until recently, no studies had directly compared ctDNA and the MCPyV antibody test (AMERK) head-to-head in the same patient population, which was a need given that clinicians must often decide which biomarker provides the most reliable signal for early intervention or de-escalation of imaging. Recent data from a multicenter study of 169 patients directly compares the predictive utility of AMERK and ctDNA tests using 703 paired samples. Both tests effectively identify patients at low risk for recurrence, as consistent with prior studies [1,12]. However, ctDNA significantly outperforms MCPyV antibody testing across several performance metrics. In this cohort, ctDNA offered significantly higher predictive utility for identifying active recurrence, with a hazard ratio of 47.9 compared to 7.3 for rising antibodies. The positive predictive value (PPV) for ctDNA at 365 days was also notably higher at 73%, whereas antibody testing reached only 52%. Notably, ctDNA remains a reliable marker during immunotherapy, where the association between rising antibodies and recurrence significantly weakens, and provides a longer median lead time to clinical detection (3.4 months vs. 2.3 months). These findings highlight ctDNA as a more universally applicable and accurate biomarker, particularly as it remains effective for the 20% of MCC cases that are virus-negative.

Simulation modeling of dual-modality surveillance further supports the primary use of ctDNA. When evaluating the utility of combining both assays, the addition of AMERK identified only one additional recurrence while introducing a substantial number of false-positive results. These findings suggest that concurrent testing does not meaningfully improve surveillance sensitivity and may lead to unnecessary clinical follow-up, reinforcing the conclusion that ctDNA can be used as a standalone biomarker for monitoring.

### 5.3. Sensitivity, Specificity, and Lead Time

In addition to the improved ability for ctDNA to detect disease recurrence compared to other blood-based markers, further potential lies in its ability to augment or even replace aspects of traditional radiographic surveillance. Standard care currently relies heavily on PET and CT scans, which have been effective at identifying gross anatomical changes but suffer from high costs and cumulative radiation exposure. Akaike et al. demonstrated that tumor-informed ctDNA is 18 times more predictive of recurrence than negative tests, with a 92% probability of remaining recurrence-free at one year if results stay negative [2]. Unlike imaging, which may miss subclinical disease, ctDNA identified 76% of recurrences with a median lead time of 2.7 months before recurrence was visible on imaging or physical examination. This lead time defines an actionable window during which clinical decisions can be meaningfully informed by molecular status (Figure 1). In fact, the concentration of ctDNA (measured in mean tumor molecules, or MTM/mL) could predict the urgency of recurrence. ctDNA levels > 10 MTM/mL, 1–10 MTM/mL, and <1 MTM/mL could predict clinical recurrence within 3 months, 6 months, and 9 months, respectively. ctDNA was less sensitive, however, in detecting superficial, low-volume cutaneous spread. However, it is important to note that ~8% of the study’s patients remained ctDNA-negative despite having a recurrence. These patients primarily had small, superficial locoregional recurrences detected by physical exam and biopsy. All in all, a negative ctDNA result and unremarkable physical examinations may provide clinicians with a high degree of confidence that a patient is truly disease-free, potentially creating a means to safely de-escalate the frequency of costly and radiation-intensive scans. However, this does not mean that rigorous physical exams should be replaced; the ctDNA assay’s reduced sensitivity for superficial, low-volume cutaneous spread means that visual inspection remains the gold standard for detecting early local recurrences.

## 6. Discussion

### 6.1. ctDNA Performance Across MCC Viral Subtypes: Biological Considerations

A clinically important question raised by the biological heterogeneity of MCC is whether ctDNA performs equivalently in virus-positive (MCPyV+) and virus-negative (UV-associated) tumors—two entities that are fundamentally distinct at the molecular, immunological, and microenvironmental levels. MCPyV-positive MCC is driven by viral oncogene expression (Large T and Small T antigens) and is characterized by an exceptionally low somatic tumor mutational burden (median 1–5 mutations/Mb), few copy number alterations, and a relatively immunosuppressed tumor microenvironment [13,14,15,16]. In contrast, virus-negative MCC carries a high UV-signature mutation burden (median ~27.5 mutations/Mb); frequent driver mutations in TP53, RB1, NOTCH1, and FAT1; numerous tumor neoantigens (reported to exceed those in melanoma and NSCLC); and a distinct tumor microenvironment with upregulated MAPK pathway activity and NK cell infiltration [14,17,18,19].

These molecular differences have meaningful implications for ctDNA assay design and performance. Tumor-informed assays derive their personalized mutation panels from tumor sequencing, and their sensitivity scales with the number of trackable somatic variants [20,21]. In virus-negative MCC, the high somatic TMB provides an abundance of unique variants from which to construct a sensitive tracking panel, theoretically favoring robust ctDNA detection [13,17]. Conversely, in virus-positive MCC, the paucity of somatic mutations (~12.5 SNVs/exome) means that fewer patient-specific variants are available for panel design, though this has not been shown to limit assay sensitivity [18].

This is evident by the Akaike et al. studies that reported high overall sensitivity (94–95%) for detecting clinically evident disease across the full MCC cohort, which was predominantly composed of virus-positive patients reflecting the ~80% prevalence of MCPyV+ tumors in the United States. Subset analyses from this cohort suggested comparable ctDNA performance between MCPyV-positive (lower somatic TMB) and MCPyV-negative (higher somatic TMB) tumors, though these data were not published given the limited subgroup sample sizes. Prospective studies formally comparing sensitivity, lead time, and NPV between viral subtypes are needed to inform surveillance de-escalation decisions—particularly in virus-positive patients, where AMERK serology provides a complementary signal, versus virus-negative patients, for whom ctDNA remains the only circulating biomarker.

### 6.2. ctDNA as a Prognostic Biomarker in MCC

The integration of ctDNA into the clinical management of Merkel cell carcinoma represents a significant shift from a generic surveillance model to one of precision oncology. Our review demonstrates that tumor-informed ctDNA assays provide a “molecular window” into disease activity that traditional imaging and viral serology often miss. The most profound clinical advantage of ctDNA is its high sensitivity (94–95%) and specificity for detecting active disease, as validated against composite clinical confirmation of recurrence (imaging, biopsy, or clinical progression per Akaike et al.), and its ability to distinguish patients based on their real-time risk of relapse [1].

The predictive power of ctDNA is particularly evident when comparing it to the existing staging system. While the AJCC stage remains a cornerstone of prognosis, it is a static measurement taken at diagnosis. In contrast, ctDNA provides a dynamic assessment of MRD. As shown by Akaike et al., the magnitude of risk associated with early ctDNA positivity suggests that molecular status may eventually supersede traditional AJCC staging for post-treatment surveillance [1]. A critical next step is validating whether the superior lead time of ctDNA allows for earlier salvage therapy and improved survival compared to traditional radiographic surveillance. For instance, Akaike et al. quantified that a positive ctDNA test identifies recurrence with a median lead time of 2.7 to 3 months before it is detectable on a PET or CT scan [1]. This lead time is crucial in an aggressive cancer like MCC, where early intervention—potentially when the tumor burden is still occult—may improve the efficacy of salvage immunotherapies.

### 6.3. ctDNA as a Guide for Therapeutic Decision-Making in MCC

Beyond its prognostic utility, ctDNA holds considerable promise as a real-time guide for therapeutic decisions in MCC, even if direct prospective evidence in this disease remains nascent. By analogy with other solid tumors—where ctDNA positivity after curative-intent treatment has already begun to inform adjuvant therapy initiation in colorectal and lung cancer—MCC clinicians may similarly use post-treatment ctDNA status to risk-stratify patients for escalation or de-escalation of adjuvant therapy. The most clinically vexing scenario is the ctDNA-positive, imaging-negative patient: current standard of care recommends observation, yet the 2.7-month lead time documented by Akaike et al. suggests an actionable window during which pre-emptive intervention warrants investigation. It is important to note that no prospective randomized trial has yet demonstrated that initiating treatment in ctDNA-positive, imaging-negative MCC patients improves survival outcomes; this strategy remains investigational and the current standard of care is active observation in this setting. As illustrated in Figure 1, emerging center-level practice is already operationalizing this framework.

ctDNA also shows early promise as a treatment response monitor during immunotherapy. Cabezas-Camarero et al. reported a case of metastatic MCC in which a rapid, near-complete decline in ctDNA tumor fraction and variant allele frequencies closely mirrored radiologic and metabolic responses after just two cycles of avelumab, positioning ctDNA as a potential real-time indicator of immunotherapy efficacy [22]. While this remains a single case report, it is conceptually consistent with findings in melanoma, where persistent ctDNA during checkpoint inhibitor therapy strongly predicts inferior outcomes [9]. Prospective trials are needed to determine whether ctDNA-guided treatment escalation, switching, or de-escalation improves outcomes in MCC.

### 6.4. ctDNA Versus Imaging in MCC

Beyond early detection, ctDNA may also serve as an alternative to frequent imaging. With a negative predictive value exceeding 93%, a series of negative ctDNA tests can provide clinicians and patients with the reassurance needed to safely de-escalate the frequency of radiation-intensive scans. This is especially vital for the approximately 20% of MCC patients with virus-negative disease, who previously had no reliable circulating biomarker for monitoring. Despite these advantages, the molecular-only nature of early ctDNA positivity, where the blood is positive but the scan is clean, remains a clinical dilemma. Currently, the standard of care remains observation until radiographic proof of disease is found.

### 6.5. Limitations and Barriers to Adoption

Despite its promise, several practical barriers currently limit the routine clinical adoption of tumor-informed ctDNA testing in MCC. First, many commercial insurers do not yet provide consistent coverage for serial ctDNA in MCC. Second, tumor-informed assay design requires tumor sequencing followed by a custom panel build, a process that typically takes 3–6 weeks. This turnaround time limits the utility of ctDNA for acute clinical decisions and requires prospective planning at the time of initial surgery. Fourth, while the negative predictive value of ctDNA has been cited at greater than 93%, this must be interpreted in the context of MCC’s approximately 40% recurrence risk: even a small proportion of false-negative results carries clinical significance in a disease where early intervention may be impactful. Notably, Akaike et al. found that approximately 8% of MCC patients with confirmed recurrence remained ctDNA-negative, predominantly those with small superficial locoregional disease. Taken together, these limitations underscore that ctDNA should currently complement established surveillance modalities, including physical examination and imaging.

## 7. Conclusions and Future Directions

In conclusion, ctDNA is a precision biomarker that addresses major unmet needs in Merkel cell carcinoma management. It provides a universal monitoring tool applicable regardless of viral status and offers superior prognostic accuracy compared to traditional viral serology. By detecting molecular recurrence months before radiographic evidence, ctDNA creates an interventional window that could redefine how we treat subclinical disease.

The inclusion of ctDNA in the 2026 NCCN Guidelines represents an important step toward its broader integration into MCC management, though routine adoption remains primarily limited to academic and specialized centers. The current evidence base consists predominantly of retrospective and observational studies, and prospective trial validation will be essential to strengthen ctDNA-guided decision-making. Moving forward, the focus must shift toward validating ctDNA-guided therapeutic escalation and de-escalation. If early initiation of therapy in ctDNA-positive, PET-negative patients is proven to be beneficial, ctDNA will not only be a tool for surveillance but a driver of a new “molecular-first” treatment paradigm in dermatologic oncology.

## Data Availability

No new data were created or analyzed in this study.
